# Variations in the Five Facets of Mindfulness in Italian Oncology Nurses according to Sex, Work Experience in Oncology, and Shift Work

**DOI:** 10.3390/healthcare12151535

**Published:** 2024-08-02

**Authors:** Elsa Vitale, Karen Avino, Rocco Mea, Maria Colomba Comes, Samantha Bove, Luana Conte, Roberto Lupo, Ivan Rubbi, Maicol Carvello, Stefano Botti, Giorgio De Nunzio, Raffaella Massafra

**Affiliations:** 1 Scientific Directorate, IRCCS Istituto Tumori “Giovanni Paolo II”, 70124 Bari, Italy; 2 Integrative Nurse Coach Academy, International Nurse Coaching Association, Miami, FL 33132, USA; kavino@inursecoach.com; 3 Cardiology Unit, San Carlo Hospital, 85100 Potenza, Italy; roccomea@yahoo.it; 4 Laboratorio di Bioinformatica e Biostatistica, IRCCS Istituto Tumori “Giovanni Paolo II”, 70124 Bari, Italy; m.c.comes@oncologico.bari.it (M.C.C.); s.bove@oncologico.bari.it (S.B.); r.massafra@oncologico.bari.it (R.M.); 5 Laboratory of Biomedical Physics and Environment, Department of Mathematics and Physics “E. De Giorgi”, University of Salento, 73100 Lecce, Italy; luana.conte@unisalento.it (L.C.); giorgio.denunzio@uisalento.it (G.D.N.); 6 Laboratory of Advanced Data Analysis for Medicine (ADAM) at the Laboratory of Interdisciplinary Research Applied to Medicine, University of Salento, Local Health Authority, 73100 Lecce, Italy; 7 “San Giuseppe da Copertino” Hospital, Local Health Authority, 73043 Copertino, Italy; roberto.lupo@uniba.it; 8 School of Nursing, University of Bologna, 48018 Faenza, Italy; ivan.rubbi2@unibo.it; 9 Community Hospital, Local Health Authority, 48013 Brisighella, Italy; maicol.carvello2@unibo.it; 10 Haematology Unit, Azienda USL-IRCCS, 42123 Reggio Emilia, Italy; stefano.botti@ausl.re.it

**Keywords:** holistic, mindfulness, oncology nursing, gender, shift work, work experience

## Abstract

Background: Oncology nurses support cancer patients in meeting their self-care needs, often neglecting their own emotions and self-care needs. This study aims to investigate the variations in the five facets of holistic mindfulness among Italian oncology nurses based on gender, work experience in oncology, and shift work. Method: A cross-sectional study was carried out in 2023 amongst all registered nurses who were employed in an oncology setting and working in Italy. Results: There were no significant differences in all five facets of holistic mindfulness (*p* ≥ 0.05) according to gender, work experience in the oncology field, and shift work. Conclusion: Could holistic mindfulness be defined as an intrinsic individual characteristic? Surely, more insights will be necessary to better define the holistic trend in oncology nursing.

## 1. Introduction

Nurses ensure high levels of practice that improve standards of care in healthcare organizations [1]. The demanding requests for quality in nursing practice may involve providing emotional support during times of extreme distress while providing therapeutic communication with patients and families, as well as successful relationships with colleagues [1,2]. In particular, the oncology field represents a stressful environment for nurses who should provide relationship-centered care in a setting where there is high exposure to death, heavy workloads, and conflictual relationship with co-workers due to the ambiguity in emotional and spiritual conditions, which increases the stress circumstances for nurses [2]. Oncology nurses help cancer patients to meet their self-care needs, often by neglecting themselves and their emotions and self-care needs [3].

According to the National Health Care Retention and RN Staffing Report, the national shifting incidence for nurses has risen to 17.2%, with a cost-related increases also detected [2]. Thus, programs have been improved to ameliorate resilience and compassion satisfaction, decreasing stress, compassion, fatigue, and burnout.

For this reason, worldwide, national, and international organizations have developed guidelines, policies, media, and education curricula to promote nurses’ well-being in relation to their self-compassion and self-care [4,5]. Several nursing organizations have suggested promoting standard levels of health and well-being in nurses by introducing interventions and strategies [6,7,8]. In this regard, an attitude that seems to be very popular thanks to its positive research is mindfulness, which embraces a holistic meditation practice associated with attention on and awareness of all dimensions of life.

The literature has suggested that nursing practices characterized by loving and kindness meditation may be more effective at ensuring satisfaction and empathy, as well as at reducing compassion fatigue and burnout, improving working environments [9,10]. Recent studies [10,11] have assessed the effectiveness of pre-shift mindfulness-based interventions at recording important decreases in stress [11], compassion fatigue, and burnout conditions.

### 1.1. Holistic Nursing

Holistic nursing is recognized as the integration of “all nursing practice that manages the total individual as its outcome” [12]. Holistic nursing has been identified as a specialty practice that focuses on reflective practices, such as meditation, and uses holistic philosophies and theories to guide practice. Holistic nurses use integrative skills to guide nurses to support people in recognizing human patterns and responses to promote wholeness. Holistic nursing deals with the protection, promotion, and optimization of health and well-being, central to healing, decreasing disease symptoms and trauma, and moderating suffering. This helps people to reach peace, well-being, agreement, and equilibrium through the treatment of human reactions to ameliorate the biopsychosocial totality of people [13].

Therefore, holistic nursing connects the body, mind, and spirit, as well as encouraging the improvement of a healthy work environment. According to the American Holistic Nurses Association [14], holistic nurses are knowledgeable in the key elements that are common with mindfulness. Thus, holistic nurses could adopt and use therapies, such as mindfulness, as an expression of the work-related stress that they might feel [15]. Holistic nursing skills could be considered to be a practice concept model related to nurses’ professional philosophies, competencies, and individual capabilities in a clinical environment [16,17]. A proactive clinical nursing perspective includes educating nurses on holistic competencies to improve work engagement or job satisfaction [18]. Education in complementary strategies and interventions used in nursing practice such as mindfulness is essential [19].

### 1.2. Mindfulness

“Mindfulness, originating from the Buddhist tradition, can be identified as a system to live one’s existence with awareness and attentiveness to the present moment with particular importance to curiosity, openness, and acceptance of all experiences without judgment” [20].

Evidence suggests that greater levels of mindfulness are linked to lower levels of depression, anxiety, and stress [21,22] and can promote well-being in nurses [23,24], with nurses ultimately experiencing a situation more coherently and reacting more effectively [25,26]. In a clinical setting, patients value healthcare professionals with greater mindfulness attitudes, resulting in better patient–clinician communication and better-quality care [27]. At the same time, mindfulness has been considered a helpful approach to improving the health and well-being of patients [27]. White [28] suggested a conceptual analysis to explain how the use of mindfulness could provide conceptual transparency to recognize its importance and adaptation in nursing. Specifically, five sub-dimensions were identified to better describe mindfulness in nursing, define as a “translational system in which the individual develops the attitude to live the present with acceptance, attention, and awareness” [29]. This particular approach may be the basic concept with practical applications needed for ensuring the well-being of nurses, as it improves therapeutic nursing actions in presence, empathy, patience for self and others, and holistic health promotion [29,30]. The result will be a positive return for the healthcare systems in the form of reduced errors and better care delivery [31,32]. However, interventions to address mindfulness in the clinical setting are still limited today [33,34,35], despite mindfulness interventions being an effective and inexpensive approach to decreasing symptoms of stress and burnout, improving quality of life satisfaction, and, consequently, ensuring better patient care [36,37,38].

### 1.3. Theoretical Framework

Nurses are considered part of the physical environment for the healing of their patients [39]. This highlights the responsibility to adopt self-care as a component of a nurse’s accountability to support holistic patient care, and this should not be considered only an individualized practice. Mariano et al. [38] highlighted the physical environment as needing the foremost holistic management of the elements suggested by Florence Nightingale, such as cleanliness, fresh air, order, nutrition, light and sunlight, warmth, writing letters for patients, being present, and attending to patient needs. Holistic nursing emphasizes self-reflection and self-care for both the nurse and the patient [40].

### 1.4. Purpose

**Figure 1:**
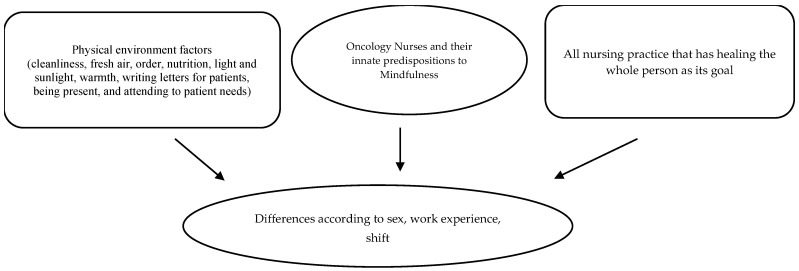
Differences in mindfulness attitudes in oncology nurses according to the physical environment and holistic nursing framework.

As shown in the Figure 1, the present study aims to focus on self-reflection on the basic sampling characteristics of Italian oncology nurses. Specifically, the research aims to investigate how the five facets of holistic mindfulness vary in Italian oncology nurses according to gender, work experience in oncology, and shift work without any interventions.

The study tested the following hypotheses:

- There is a difference in nurses’ mindfulness attitudes according to gender;
- There is a difference in the mindfulness attitudes of nurses according to work experience in oncology settings;
- There is a difference in nurses’ mindfulness attitudes according to their shift patterns.

## 2. Materials and Methods

### 2.1. Study Design

This cross-sectional survey study was carried out in 2023 among all registered nurses employed in an oncology setting in Italy.

### 2.2. Study Size

In 2022, approximately 395,000 nurses were employed in the Italian Healthcare System. Of these, 277,171 nurses were employed in the National Healthcare Service. However, the exact number of nurses employed in an oncology setting is unknown. Therefore, it was not possible to precisely assess the sample size.

In 2021, the Italian Ministry of Health stated that about 59.2% of Italian healthcare professionals (*n* = 617,246) [41] were employed as nurses. Considering the Miller and Brewer formula [42] and fixing the confidence interval at 95%, the representative sample size assessed was 400 nurses including all the nursing disciplines. Taking into consideration the fact that in the Italian Healthcare System, there are nearly 70 clinical specialties [43], we could also assume as representative a sample size reaching at least half the sample size calculated.

### 2.3. Questionnnaire Administration

A Google Moduli questionnaire was performed and disseminated via the “Nurseallface” social media page by inviting nurses employed in the oncology field to complete the questionnaire. All visitors could access the presentation letter of the study, but only those who gave their consent to participate and declared themselves to be an oncology nurse could proceed further with the questionnaire.

### 2.4. Questionnaire Items

The questionnaire contained some demographic questions, such as the following:

- Gender: female, male, or not answered;
- Age: under 30 years, 31–40 years, 41–50 years, and over 51 years,
- Years of work experience as a nurse: under 5 years, 6–10 years, 11–15 years, 16–20 years, 21–30 years, and 31–40 years;
- Years of work experience in the oncology field: under 5 years, 6–10 years, 11–15 years, 16–20 years, 21–30 years, and 31–40 years;
- Shift work: one shift per day (morning), two shifts per day (morning and afternoon), and three shifts per day (morning, afternoon, and night).

The Five Facet Mindfulness Questionnaire (FFMQ) [44,45] assesses mindfulness as a whole psychological frame with the following five essential sub-dimensions:

- “Observe” is the ability to monitor stimuli coming from your own body;
- “Describe” is the attitude to verbally identify those stimuli;
- “Acting with Awareness” or “Acta-ware” is the ability to pay attention to activities performed in the present moment without mechanically performing them;
- “Non-judging” is the ability to not express judgment;
- “Non-reacting” is the ability to not proceed automatically without thinking.

The FFMQ was validated in terms of its content validity to provide stable sub-dimensions of mindfulness thanks to several psychometric networks [46]. The FFMQ contains 39 self-reported items associated with a Likert scale, ranging from 1, defined as “totally disagree” to 5, defined as “totally agree” [46]. Item numbers 3, 5, 8, 10, 12, 13, 14, 16, 17, 18, 22, 23, 25, 28, 30, 34, 35, 38, and 39 were considered to be reversed in their scoring. By summing the scores for each sub-dimension, a number was obtained, which indicated a higher tendency in the specific sub-dimension. The FFMQ was also validated in Italian [47], reporting α-Cronbach = 0.83, representing an expression of good internal consistency [46,47]. Additionally, all items contributed to the level of homogeneity of the scale since no exclusion leads to higher alpha values.

### 2.5. Data Analysis

Data were collected in an Excel data sheet and then processed. All demographic characteristics such as gender, work experience in the oncology field, and shift work were considered as categorical variables, and the FFM questionnaire and its related sub-dimensions were considered continuous ones. ANOVA tests were performed to highlight differences between the five sub-dimensions of the FFM questionnaire according to gender, work experience in the oncology field, and shift work. All *p*-values less than 0.05 were considered statistically significant.

### 2.6. Ethical Considerations

According to the Committee on Publication Ethics (COPE), the questionnaire was anonymous. At the beginning of the questionnaire, a clear explanation of this study and its purpose was provided. It was emphasized that participation was voluntary and that the participant could withdraw from this study at any time. Immediately after the study submission letter was drafted, a mandatory clause on consent to participate in this study was introduced. If the participant gave the own consent to participate, the entire questionnaire appeared, and the participant could read it and decide whether or not to give the answers and submit them. No data or alpha-numerical codes were posted to guarantee the anonymity of the participants. Concerning the competencies and functions of the Italian Ethical Committee (EC) [48], the EC expresses opinions on the following types of studies: the protocols of clinical drug trials, observational clinical trials, and clinical trials with medical devices; the protocols for the therapeutic use of investigational drugs outside clinical trials or for biomedical, psycho-educational, social, or other research involving human subjects; epidemiological, evaluative, and medico-social research projects that require the collection of personal data with environmental ethics implications; patient information sheets and informed consent forms; ethical–scientific, methodological, and economic aspects of experimental research protocols or amendments; and the qualification of investigators to conduct the proposed research, as well as the ethical and scientific aspects of the same research. Since the present study explored mindfulness attitudes among Italian oncology nurses according to gender, work experience in the oncology field, and shift work, without investigating the above-mentioned fields of research, the EC’s approval was not needed. At the beginning of the questionnaire, a clear explanation of this study and the purpose of it was clearly provided. Participants who gave their written consent to participate in this study were allowed to move on to later questions, and their data were collected. Conversely, participants who did not give their consent were unable to continue to answer the questionnaire.

## 3. Results

### Nurses’ Demographic Characteristics

**Figure 2:**
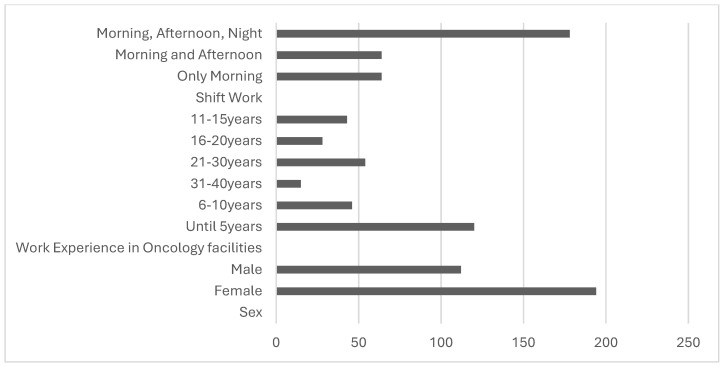
Sampling characteristics among oncology nurses.

A total of 306 Italian oncology nurses agreed to participate in the present study. Of these, 194 were females and 112 were males. In total, 90 nurses aged less than 30 years, 85 less between 31 and 40 years, 68 nurses aged between 41 and 50 years, and 63 nurses aged over 51 years participated. Globally, considering the total number of years of work experience, 97 nurses worked less than 5 years, 50 nurses were employed for between 6 and 10 years, 34 nurses worked between 11 and 15 years, 32 nurses were employed for between 16 and 20 years, 60 nurses worked between 21 and 30 years, and 33 nurses were employed for between 31 and 40 years. Most of the enrolled nurses were also employed during the night shift (*n* = 178), and the others were equally divided into the only morning shift and the afternoon shift (Figure 2).

Differences in the five facets of mindfulness according to gender, oncology work experience, and shift work were studied.

Considering the gender characteristic, there were no significant differences in all five facets of mindfulness (*p* ≥ 0.05), as shown in the Table 1.

Considering oncology work experience, there were no significant differences in all the five facets of mindfulness (*p* ≥ 0.05), as shown in the Table 2.

Considering shift work, there were no significant differences in all five facets of mindfulness (*p* ≥ 0.05), as shown in the Table 3.

## 4. Discussion

The present study aimed to focus on self-reflection on the basic sampling characteristics of Italian oncology nurses. Specifically, the research aimed to investigate how the five facets of holistic mindfulness varied in Italian oncology nurses according to gender, work experience in oncology, and shift work without any interventions.

Our findings showed no significant differences in all the five facets of mindfulness (*p* ≥ 0.05) in all the sampling characteristics considered.

### 4.1. Benefits for Nurses in Mindfulness Attitudes

Previous studies have explained that mindfulness moderated the progression of anxiety, depression, and stress in oncology healthcare professionals [49,50], and it also reduced negative thoughts [51] and emotional exhaustion and fatigue [52], had a positive effect on psychological well-being in nurses [53,54,55,56], and led to improvements in attention [57] and problem-solving ability [58]. Therefore, mindfulness may be considered a positive and proactive element in oncology nurses, whose tasks include the management of complex diseases with poor prognosis, severe pain, and distress. The deaths witnessed and difficulties in handling patient and family situations negatively impacted on job satisfaction, stress, and burnout [59,60,61], resulting in oncology nursing being the field most influenced by the nursing shortage [62,63]. In this regard, nurses who recorded higher scores of mindfulness could be more attentive to patient requirements and helpful in their related problems [64] and experience less stress due to having a favorable awareness of the present moment [65]. Therefore, nurses who recorded innately higher levels of mindfulness attitudes compared to nurses who did not more actively participated in the patient care process [66]. Mindfulness also supported the influence of nurses in creating a healing physical environment [50], highlighting the importance of self-care as a holistic mode to promote the health and well-being of the patient. Mindfulness-based interventions were the most frequent and validated interventions implemented in healthcare organizations to better address stress in several healthcare contexts associated with emotion, adaptation, and regulation [67]. According to our findings, another study showed that in older and younger nurses, mindfulness scores were not significantly different [68]. It may be possible that this attitude overlapped with the nurses’ sense of emotional equilibrium, which misunderstood the expected psychosocial care needs [68]. Additionally, in the oncology environment, the complexity of psychosocial care required nurses to handle different typologies of emotions among patients and their families, which, in turn, had a negative impact on the reactions and emotions of nurses [69].

### 4.2. Mindfulness and Emotion Regulation

Nurses who recorded mindfulness characteristics, such as non-judgmental awareness and acceptance of the present moment’s experience, were less inclined to suppress their emotions [69]. In 2012 [70], as study highlighted a correlation between mindfulness and emotion regulation improvement. Additionally, ref. [71] reported that mindfulness-trained individuals better managed their emotions in their working environments. In this regard, a healthy work environment among nurses appeared to be important for patient goals, staff gratification, and improved quality of care, since numerous nurses believed that they were more accomplished in using their understanding of a patient’s decision-making to improve end-of-life care, with mindfulness supplying training on alternatives to care. Oncology nurses could benefit from adopting mindfulness approaches to better understand their emotions and ideas, share their personal circumstances, and improve support systems to provide holistic care without implicating their ethics [72].

### 4.3. Strengths and Limitations

This study had some limitations since it was a cross-sectional study and the questionnaire was administered online. Additionally, the sample size of participants was a convenience sample since it included only nurses who voluntarily decided to participate. Therefore, findings were attributed to causal interpretations of the relationships obtained. Additionally, the questionnaire was a self-reported tool without any possibilities for recalling the bias in and social desirability of responding. Future research could explore the associations between mindfulness and emotional suppression associated with nurses’ characteristics, as well as investigate all possible associations between mindfulness, emotional regulation, and other aspects, such as the coping style of nurses, supportive relationships with mindful awareness, and non-judgmental acceptance.

## 5. Conclusions

Our findings showed no significant differences in all five facets of mindfulness (*p* ≥ 0.05) in all the sampling characteristics considered. Thus, future longitudinal studies that further explore the impact of mindfulness on nurse well-being and patient care outcomes will be addressed to better highlight all the abovementioned aspects. This may have implications for the use of mindfulness practice, with a focus on emotion regulation and having self-compassion for others (loving kindness meditation), supported by implementation intentions or planning for meditation. Reducing fatigue in oncology nurses has benefits for a nurse’s emotional well-being and for the safety of and compassionate care provided to patients.

The initial aim of our study was to investigate whether the five subdimensions of mindfulness varied among oncology nurses regarding their characteristics, such as gender, years of work experience, and shift work. This study’s results showed acceptable levels of mindfulness in all five sub-dimensions investigated. However, these dimensions did not vary according to the aforementioned characteristics. Therefore, we could conclude that mindfulness may be an intrinsic individual characteristic. Surely, more insights will be necessary to better define this trend in oncology nursing, since the literature suggests that mindfulness is a helpful approach to improve functional emotional regulation based on environmental conditions, such as non-willing patients and communication with the families of patients and nursing managers and physicians, especially in the field of oncology, with pain and emotions being associated with shift work [73].

## Figures and Tables

**Table 1:** Differences in the five facets of mindfulness according to gender.

The Five Facets of Mindfulness		Mean	Standard Deviation	C.I. 95%		F	*p*
				Min.	Max		
Observing	Female	29.2371	5.25174	28.4934	29.9808	0.026	0.872
	Male	29.3393	5.52399	28.3050	30.3736		
	Total	29.2745	5.34420	28.6733	29.8757		
Describing	Female	24.7732	6.68616	23.8264	25.7200	1.316	0.252
	Male	25.5893	4.54914	24.7375	26.4411		
	Total	25.0719	5.99793	24.3972	25.7466		
Acting	Female	29.4794	6.57299	28.5486	30.4102	0.402	0.526
	Male	29.0268	4.88878	28.1114	29.9422		
	Total	29.3137	6.00707	28.6380	29.9895		
Non-judging	Female	25.2268	4.06681	24.6509	25.8027	1.045	0.307
	Male	24.7589	3.45954	24.1112	25.4067		
	Total	25.0556	3.85646	24.6217	25.4894		
Non-reactivity	Female	19.2165	4.44244	18.5874	19.8456	1.609	0.206
	Male	18.5446	4.49802	17.7024	19.3869		
	Total	18.9706	4.46727	18.4681	19.4731		

C.I.: Confidence interval; *p* ≤ 0.05 is statistically significant.

**Table 2:** Differences in the five facets of mindfulness according to oncology work experience.

The Five Facets of Mindfulness		Mean	Standard Deviation	C.I. 95%		F	*p*
				Min.	Max		
Observing	Until 5 y	29.2167	5.56578	28.2106	30.2227	0.129	0.986
	Until 10 y	29.0652	5.67020	27.3814	30.7491		
	Until 15 y	29.7442	4.90914	28.2334	31.2550		
	Until 20 y	29.2857	4.64963	27.4828	31.0887		
	Until 30 y	29.0556	5.61473	27.5230	30.5881		
	Until 40 y	29.8000	4.57009	27.2692	32.3308		
	Total	29.2745	5.34420	28.6733	29.8757		
Describing	Until 5 y	25.2750	5.85822	24.2161	26.3339	0.927	0.464
	Until 10 y	25.0870	6.47328	23.1646	27.0093		
	Until 15 y	24.3256	5.52794	22.6243	26.0268		
	Until 20 y	23.7143	6.82510	21.0678	26.3608		
	Until 30 y	26.2037	5.57074	24.6832	27.7242		
	Until 40 y	24.0000	6.81385	20.2266	27.7734		
	Total	25.0719	5.99793	24.3972	25.7466		
Acting	Until 5 y	29.4167	5.55429	28.4127	30.4206	0.815	0.539
	Until 10 y	29.8913	9.04121	27.2064	32.5762		
	Until 15 y	28.3488	4.89841	26.8413	29.8563		
	Until 20 y	28.2500	5.04517	26.2937	30.2063		
	Until 30 y	30.2037	5.12993	28.8035	31.6039		
	Until 40 y	28.2667	5.47027	25.2373	31.2960		
	Total	29.3137	6.00707	28.6380	29.9895		
Non-judging	Until 5 y	24.9750	3.98413	24.2548	25.6952	0.686	0.634
	Until 10 y	25.3913	3.46717	24.3617	26.4209		
	Until 15 y	24.4884	3.76938	23.3283	25.6484		
	Until 20 y	24.6429	3.60262	23.2459	26.0398		
	Until 30 y	25.2593	3.96297	24.1776	26.3409		
	Until 40 y	26.3333	4.43471	23.8775	28.7892		
	Total	25.0556	3.85646	24.6217	25.4894		
Non-reacting	Until 5 y	18.7083	4.60251	17.8764	19.5403	0.981	0.429
	Until 10 y	19.3913	3.75635	18.2758	20.5068		
	Until 15 y	19.7907	4.38373	18.4416	21.1398		
	Until 20 y	19.6786	4.02817	18.1166	21.2405		
	Until 30 y	18.1296	4.65426	16.8593	19.4000		
	Until 40 y	19.1333	5.60442	16.0297	22.2370		
	Total	18.9706	4.46727	18.4681	19.4731		

C.I.: Confidence interval; *p* ≤ 0.05 is statistical significant.

**Table 3:** Differences in the five facet of mindfulness according to shift work.

The Five Facets of Mindfulness		Mean	Standard Deviation	C.I. 95%		F	*p*
				Min.	Max		
Observing	One shift	29.6719	5.14875	28.3858	30.9580	0.656	0.520
	Two shifts	29.7031	5.09179	28.4312	30.9750		
	Three shifts	28.9775	5.50804	28.1628	29.7923		
	Total	29.2745	5.34420	28.6733	29.8757		
Describing	One shift	25.9531	5.96931	24.4620	27.4442	1.008	0.366
	Two shifts	25.1719	4.80923	23.9706	26.3732		
	Three shifts	24.7191	6.37702	23.7758	25.6624		
	Total	25.0719	5.99793	24.3972	25.7466		
Acting	One shift	30.0156	5.05366	28.7533	31.2780	0.779	0.460
	Two shifts	29.5625	8.05512	27.5504	31.5746		
	Three shifts	28.9719	5.44768	28.1661	29.7777		
	Total	29.3137	6.00707	28.6380	29.9895		
Non-judging	One shift	25.2813	3.94594	24.2956	26.2669	0.857	0.425
	Two shifts	24.5000	3.52767	23.6188	25.3812		
	Three shifts	25.1742	3.93851	24.5916	25.7567		
	Total	25.0556	3.85646	24.6217	25.4894		
Non-reacting	One shift	18.7969	4.39039	17.7002	19.8936	0.079	0.924
	Two shifts	19.1094	3.93647	18.1261	20.0927		
	Three shifts	18.9831	4.68978	18.2894	19.6768		
	Total	18.9706	4.46727	18.4681	19.4731		

C.I.: Confidence interval; *p* ≤ 0.05 is statistical significant.

## Data Availability

Data are available from the corresponding author upon reasonable request.
